# Separation and detection of D-/L-serine by conventional HPLC

**DOI:** 10.1016/j.mex.2022.101752

**Published:** 2022-06-17

**Authors:** Hiroki Shikanai, Kazuko Ikimura, Momoko Miura, Tsugumi Shindo, Akane Watarai, Takeshi Izumi

**Affiliations:** a Department of Pharmacology, Faculty of Pharmaceutical Sciences, Health Science University of Hokkaido, Japan; b Advanced Research Promotion Center, Health Science University of Hokkaido, Japan; c Eicom Corporation, Research and Development, Japan; d School of Pharmaceutical Sciences, Health Sciences University of Hokkaido, Japan

**Keywords:** D-amino acid, Detection, Diastereomer method, D-/L-serine, N-acetyl-L-cysteine (NAC), Ortho-phthalaldehyde (OPA), Reversed phase HPLC with ECD, Separation

## Abstract

D-serine has a role as an endogenous allosteric agonist of N-methyl-D-aspartate (NMDA) receptor in the mammalian brain. In this study, we present a detailed description of our method that measures D-/L-serine by using conventional high performance liquid chromatography (HPLC).

• We reacted D-serine and L-serine with ortho-phthalaldehyde (OPA) and N-acetyl-L-cysteine (NAC) to form diastereomeric isoindole derivatives, then we separated and detected them by conventional reversed phase HPLC with electrochemical detector (ECD).

• We present typical measurement data of rat brain homogenate as an example of a convenient, appropriate method for measuring brain concentrations of D-serine.

• Since many peaks appear in biological samples, we confirmed that the peaks were derived from serine by treating the sample with D-amino oxidase and catalase to decompose D-serine. As a results, one peak disappeared, suggesting that it is derived from D-serine.

## Graphical abstract

**Figure:**
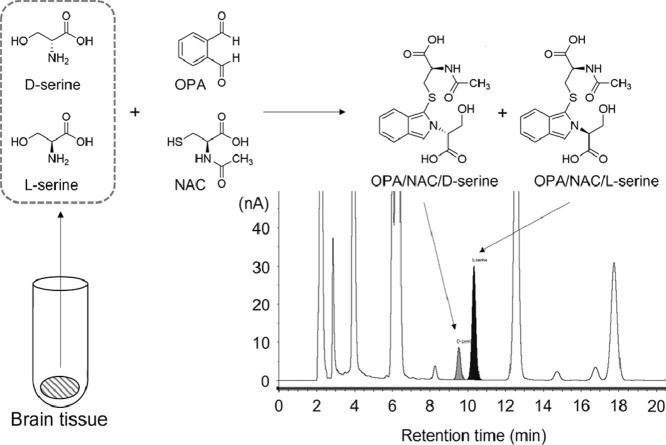

**SPECIFICATIONS TABLE**

**Table:** 

Subject Area;	Biochemistry, Genetics and Molecular Biology
More specific subject area;	Bioanalysis, analytical chemistry
Method name;	Separation and detection of D-/L-serine by conventional reversed phase HPLC with ECD
Name and reference of original method;	N/A
Resource availability;	A manual of D-Amino Acid (D-serine, L-serine) measurement using HPLC from the homepage of Eicom Corporation: https://7a929834-6b18-462b-87933bfdb35b03b6.filesusr.com/ugd/d511e8_83a4b6a872654cc6b5339d0eaeebc7ab.pdf

## Introduction

D-serine is the D-isomer of L-serine, which is one of the major amino acids that make up proteins. D-serine is present in high levels in the mammalian brain, and has a role as an endogenous allosteric agonist of N-methyl-D-aspartate (NMDA) receptor [1, 2]. Therefore, the pharmacological manipulation of D-serine signaling is expected to be a therapeutic tool for psychiatric disorders [3]. The main source of D-serine in the brain is conversion from L-serine by serine racemase, which is abundant in the brain [3]. D-serine and L-serine are in an enantiomer relationship, and a special method must be used for separation and detection. One useful method for enantiomer separation is a chiral high performance liquid chromatography (HPLC) method, using an HPLC with chiral stationary phase [4], though the expense of the columns is a limitation of this method. Another method is the diastereomer method, which converts enantiomers to diastereomers by reacting them with chiral derivatizing agents and then separates them by normal HPLC [5]. Most measurements in the diastereomer method use fluorescent chiral reagents for diastereomer formation, followed by HPLC on silica gel or reversed-phase HPLC for separation, and ultra violet detector (UV) for detection [6]. In this study, we reacted D-serine and L-serine with ortho-phthalaldehyde (OPA) and N-acetyl-L-cysteine (NAC) to form diastereomeric isoindole derivatives, then separated and detected them by conventional reversed phase HPLC with electrochemical detector (ECD) [7]. We present a detailed description of our method and typical measurement data of rat brain homogenate as an example of a convenient, appropriate method for measuring brain concentrations of D-serine.

## Method details

### Chemicals

Chemicals were purchased from the following companies: sodium dihydrogenphosphate dihydrate (NaH_2_PO_4_•2H_2_O; Cat. No. 84190-03019), disodium hydrogenphosphate 12-water (Na_2_HPO_4_•12H_2_O; Cat. No. 84125-0301) and potassium carbonate (K_2_CO_3_; Cat. No. 43250-0301) from JUNSEI (Tokyo, Japan); ethylenediaminetetraacetic acid (EDTA)-2Na (Cat. No. 343-01861), glycine (Cat. No. 073-00737), ethanol (Cat. No. 057-00451), catalase (Cat. No. 035-12903), 2-Amino-2-hydroxymethyl-1,3-propanediol (Tris; Cat. No. 207-06275) and 2 mol hydrogen chloride (HCl; Cat. No. 083-02715) obtained from FUJIFILM Wako (Osaka, Japan); D-serine (Cat. No. S0033), L-serine (Cat. No. S0035) and ortho-phthalaldehyde (OPA; Cat. No. P0280) obtained from TCI (Tokyo, Japan); D-amino acid oxidase (DAO; Cat. No. DOX2) was obtained from Biozyme Laboratories (South Wales, UK); and N-acetyl-L-cysteine (NAC; Cat. No. 00512-84) and HPLC-grade methanol (Cat. No. 21929-23) were obtained from nacalai tesque (Kyoto, Japan). All of the above chemicals were analytical grade, except the methanol.

### Animals

Male Wistar Kyoto (WKY/Ezo) rats were bred in our laboratory, and male Siz:Wistar rats were purchased from Shimizu Laboratory Materials Co., Ltd. (Kyoto, Japan). 6-week-old WKY/Ezo rats were used for the experiment to simply measure D-/L-serine, and 8-week-old Siz:Wistar rats were used for the D-serine removal experiment. Rats were housed in a room with a 12-h light-dark cycle under a constant temperature (21±2°C). All animal procedures were performed under the Guidelines of National Institutes of Health Guidelines for the Care and Use of Laboratory Animals.

### Sample preparation

Rats were sacrificed and their brains were dissected and sliced into 2 mm-thick coronal sections using a brain slicer (Muromachi, Japan) on ice. The prefrontal cortex (PFC) containing medial prefrontal cortex area (2.60-4.60 mm anterior to the bregma) was dissected out according to the brain atlas. To measure amino acid contents, samples were crushed on ice using a Polytron homogenizer (PT1200E, Kinematica AG, Malters, Switzerland) at full speed with 3 consecutive 10 second bursts in 10 v/v% methanol solution, which was 10 times the wet weight of the tissue. After incubation on ice for 15 min, samples were centrifuged at 15,000 × *g* for 15 min (4°C). The supernatant was passed through a 0.22 μm filter (Millex-GV, Merck, Darmstadt, Germany), aliquoted into a separate microcentrifuge tube, and diluted 50-fold with 10 v/v% methanol solution. In the D-serine removal experiment, the sample was diluted 10-fold with 0.2 M Tris buffer (pH 8.3) containing 4 unit/mL D-amino acid oxidase and 2,000 unit/mL catalase for 30 minutes at room temperature. After the reaction, the mixture was filtered through an ultrafiltration membrane (MWCO=10,000 Da).

### Derivatization

For measurement, samples were derivatized by mixing with 4 mM OPA-NAC solution at a ratio of 4:1 and reacting for 150 s at room temperature. A portion (10−20 μL) of the reaction mixture was injected onto high-performance liquid chromatography (HPLC) with the electrochemical detector (ECD). D-serine, L-serine and glycine in the sample react with OPA-NAC solution, and form diastereomeric isoindole derivatives that are separable by reversed phase HPLC.

### HPLC-ECD

The analyses were carried out using HPLC-ECD system (Eicom, Kyoto, Japan) which consisted of a chromatograph (EP-300, flow rate: 500 μL/min), a degasser (DG-300), a column oven (ATC-300), a precolumn (CA-ODS), a separation column (Eicompak EX-3ODS, ODS (C18) column, 4.6 mm i.d. × 100 mm with 3 µm particles, column temperature 30°C), a working glassy carbon electrode (WE-GC), an ECD (ECD-300) with an applied voltage of +600 mV vs. Ag/AgCl, and a data recording system (EPC-500 controlled by PowerChrom software 2.5). The mobile phase was a mixture of 0.1 M phosphate buffer (pH 6.0) and methanol at a ratio of 82:18 including EDTA-2Na (5 mg/L).

### Mobile phase

Mobile phase was prepared in the following manner.

1 Solution A: a total of 15.6 g of sodium dihydrogen phosphate dihydrate (NaH_2_PO_4_•2H_2_O) was dissolved in 1,000 mL of water.
2 Solution B: a total of 35.8 g of disodium hydrogen phosphate 12-water (Na_2_HPO_4_•12H_2_O) was dissolved in 1,000 mL of water.
3 100 mmol/L phosphate buffer (pH 6.0): 1,000 mL of Solution A and 160 mL of Solution B were mixed.
4 Mobile Phase: 820 mL of 100 mmol/L phosphate buffer (pH 6.0) and 180 mL of methanol were mixed, and 1 mL of 5 mg/mL EDTA-2Na solution was added.

### Derivatization reagent

Derivatization reagent was prepared in the following manner.

1 0.5 mol/L carbonate buffer (pH 10): 6.9 g of potassium carbonate (K_2_CO_3_) was dissolved in approximately 50 mL of water, after which 10 mL of 2 N hydrogen chloride (HCl) and water were added to 100 mL.
2 20 mmol/L OPA-NAC solution: 27 mg of OPA was dissolved in 1 mL of ethanol, after which 32.7 mg of NAC and 0.5 mol/L carbonate buffer (pH 10) were added to 10 mL.
3 4 mmol/L OPA-NAC solution: 20 mmol/L OPA-NAC solution was diluted 4 times with 0.5 mol/L carbonate buffer (pH 10).

### Standard solutions

10 mmol/L L-serine/D-serine/glycine were prepared in 10 v/v% methanol and stored at -20°C or lower. Stock solution was diluted with 10 v/v% methanol to required concentration before use. All standard solutions were stored in plastic containers to prevent adsorption to glassware.

## Representative results

**Fig. 1:**
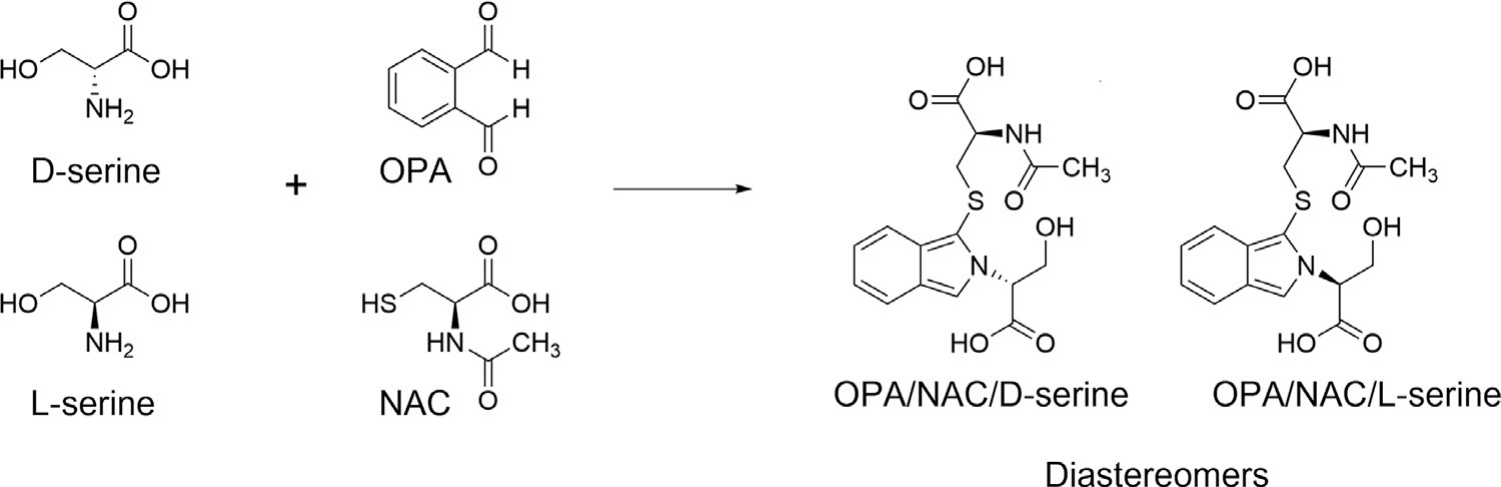
Derivatization of D-/L-serine with OPA and NAC.

In contrast to D-serine and L-serine which are present as pairs of enantiomers, derivatization of D-/L-serine with OPA and NAC (OPA/NAC/D-/L-serine) have two stereogenic centers in their structure, and therefore exist as a mixture of pairs of diastereomers. These diastereomers have a stereogenic center arising from serine, in the carbon atom α to the amine (D or L to serine), and also a stereogenic center derived from NAC (L to NAC) (Fig. 1).

**Fig. 2:**
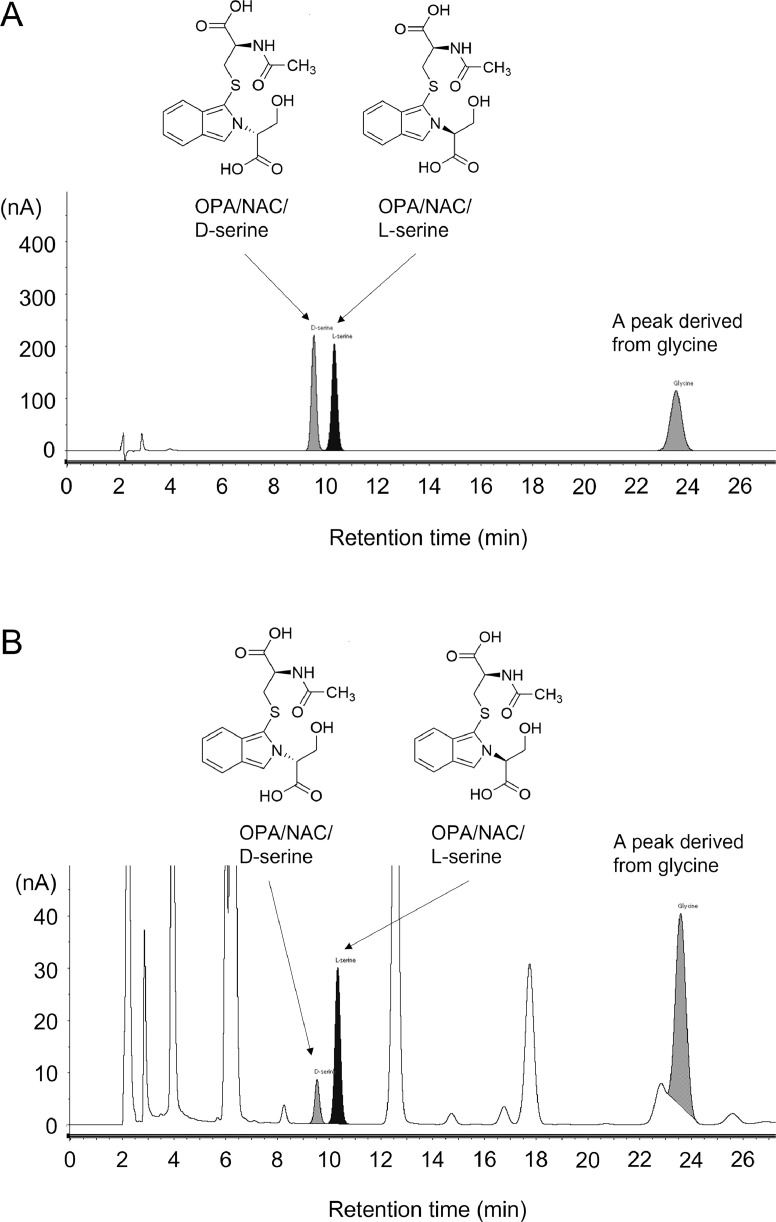
HPLC-ECD chromatograms for the analytical resolution of diastereomers derived from D-/L-serine. (A) Chromatogram obtained with 10 μmol/L D-/L-serine and glycine standard mixture (10 μL injection). The resolution between diastereomers (R) = 2.145 (B) Chromatogram obtained with 1.6 mg tissue/mL rat brain (mPFC) homogenate (10 μL injection). R = 2.145

An equimolar mixture of OPA/NAC/D-serine and OPA/NAC/L-serine was used to ascertain the analytical conditions required to achieve a good separation of these diastereomers. Several HPLC columns, pHs and mobile phases were examined; we achieved good separation and detection of these diastereomers with the reverse-phase ODS (C18) column and ECD under our experimental conditions (Fig. 2A). The resolution between diastereomers (R) = 2.145

**Fig. 3:**
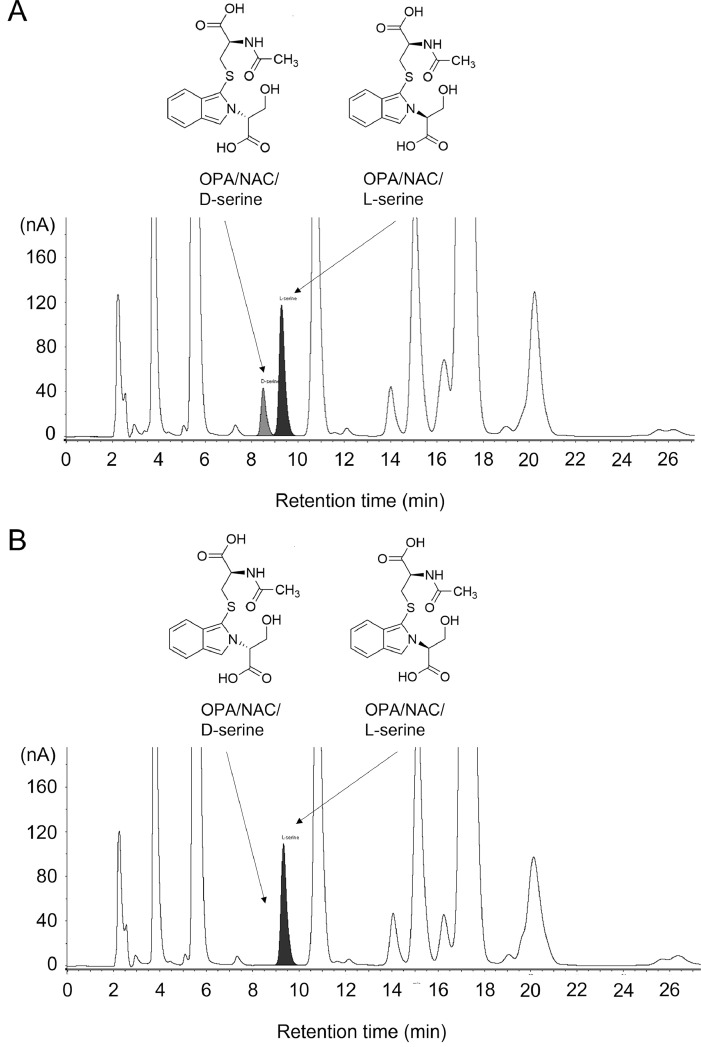
HPLC-ECD chromatograms for the D-serine removal experiment. (A) Chromatogram obtained with 1.6 mg tissue/mL rat brain (cerebral cortex) homogenate (10 μL injection). (B) Chromatogram obtained with 1.6 mg tissue/mL rat brain (cerebral cortex) homogenate treated with D-amino acid oxidase and catalase (10 μL injection).

**Fig. 4:**
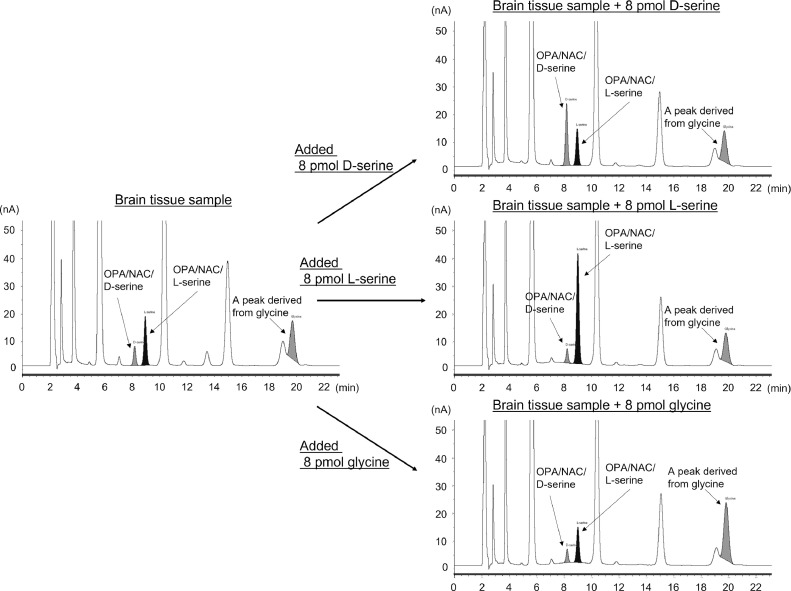
HPLC-ECD chromatograms for the exogenous D-serine, L-serine, and glycine addition experiment. Addition of these (8 pmol each) to the sample increased the peaks that seem to be derived from D-serine, L-serine, and glycine.

Next, we injected a homogenized sample of rat cerebral cortex into HPLC under similar conditions, and two peaks were identified in the retention time (T_R_) of OPA/NAC/D-serine (T_R_ = 9.54) and OPA/NAC/L-serine (T_R_ = 10.34) (R = 2.145) (Fig. 2B). Since many peaks appear in biological samples, we confirmed that the peaks were derived from serine by treating the sample with D-amino oxidase and catalase to decompose D-serine. As a results, a peak disappeared, suggesting that it is derived from D-serine (Fig. 3). This revealed that the two peaks were OPA/NAC/D-serine and OPA/NAC/L-serine, and showed that good separation was also achieved in the biological sample. Moreover, addition of exogenous D-serine, L-serine, and glycine (8 pmol each) to the sample increased the peaks that seem to be derived from D-serine, L-serine, and glycine (Fig. 4), also suggesting that the peak identification was correct.

Meanwhile, standards were injected repeatedly 3 times at 30-min intervals, but little variation between measurements within the range of 60 min was observed (peak ratio of 1st: 2nd: and 3rd measurement of D-serine-derived peak = 1:0.98:0.97, that of L-serine-derived peak = 1:1.03:1.03). In addition, standards were injected twice with an interval of 10 hours, but the measurements did not change (peak ratio of 1st: 2nd measurement of D-serine-derived peak = 1:1.01, that of L-serine-derived peak = 1:1.00). Moreover, the injection amounts and measured values of D-serine, L-serine and glycine were linear within the range of the measured values of the sample (r value of correlation analysis = 0.99, 0.97, and 1.00, respectively). These results support the stability of our measurement system.

## Discussion

In the present study, we measured the values in the PFC of rats and found 200–250 pmol/mg tissue for D-serine, 950–1000 pmol/mg tissue for L-serine, and a DL ratio (D-serine/D-serine + L-serine) of 0.19–0.24. Table 1 shows the results of previous studies in which D-serine was measured in the rodent brain (if the range of measured values and DL ratio are not specified in a study, the maximum possible range was estimated based on the measured values in the study). Our measurement results for D-serine (Shindo et al., 2022) are mostly in agreement with that in Hashimoto et al., (1992) [8]. The D-serine values in Morikawa et al., (2001) [9] and in Savignac et al., (2013) [10] are slightly different from our values, but in the same order range. In contrast, the D-serine values in Song et al., (2008) are one order larger than ours [11]. Measured D-serine absolute values may be different between studies due to differing animal species, site and method of sampling, and measurement method. However, the DL ratio values are almost the same between studies at around 0.2 except in Morikawa et al., (2001); this may be related to the difference in animal species (rats versus mice). Thus, it is reasonable to compare the dynamics of D-/L-serine between different studies using the DL ratio [12]. The method we report here is convenient to implement and the distribution in DL ratio is small. We strongly hope that this method can contribute to the study of serine in the central nervous system.

**Table 1:** Previous studies of D-/L-serine contents and DL ratio in rodent brains.

Reference	Analytical method	D-serine	L-serine	DL ratio (D-serine/ D-serine + L-serine)
		(pmol/mg tissue)		
Shindo et al., (2022) *WKY rats, mPFC*[7]	HPLC-ECD	252.2 ± 20.1	1003.4 ± 74.4	0.20 ± 0.00
Hashimoto et al., (1992) *Wistar rats, brain*[8]	HPLC-FD	220 ± 4^b^	660 ± 11^b^	0.25 ± 0.01^b^
Morikawa et al., (2001) *ddY mice, cerebrum*^a^[9]	HPLC-FD	423.2 ± 21.5	(data not shown)	0.329
Savignac et al., (2013) *S.D. rats, frontal cortex*[10]	HPLC-FD	40.3 ± 2.9	141.4 ± 13.6	0.19–0.25^b^
Song et al., (2008) *S.D. rats, corte*x [11]	HPLC-MS/MS	971–4570^b^	(data not shown)	0.184–0.239

Wistar Kyoto rats, WKY rats; medial prefrontal cortex, mPFC; electrochemical detector, ECD; fluorimetric detection, FD; Sprague-Dawley rats, S.D. rats; tandem mass spectrometer, MS/MS.

a The cerebrum means a large area of the brain containing the cerebral cortex.

b These values were estimated based on the measured values in each reference.

## Declaration of Competing Interests

The authors declare that they have no known competing financial interests or personal relationships that could have appeared to influence the work reported in this paper.
